# Evaluation of the correlation between sleep quality and work engagement among nurses in Shanghai during the post‐epidemic era

**DOI:** 10.1002/nop2.1735

**Published:** 2023-04-10

**Authors:** Zhuang Hui‐ren, Ma Li‐li, Liu Qin, Zhang Wei‐ying, Yu Hai‐ping, Zhao Wei

**Affiliations:** ^1^ Shanghai East Hospital, Tongji University School of Medicine Shanghai China; ^2^ Department of Nursing Health School Attached to Shanghai University of Medicine & Health Sciences Shanghai China; ^3^ Suzhou Science & Technology Town Hospital Tongji University School of Medicine Shanghai China

**Keywords:** COVID‐19, nurse, sleep quality, work engagement

## Abstract

**Aim:**

To examine the status quo and influencing factors of sleep quality and work engagement of nurses participating in COVID‐19 during the post‐epidemic era and to study the relationship between them.

**Design:**

We conducted a cross‐sectional survey and correlational and predictive logic to determine the association between sleep quality and work engagement among nurses in Shanghai during the post‐epidemic era.

**Methods:**

This design involved 1060 frontline nurses in Shanghai. The Pittsburgh Sleep Quality Index questionnaire and the Utrecht Work Engagement Scale‐9 scales were used for data collection.

**Results:**

This study found that the sleep quality of frontline nurses was impaired and the nurses with poor sleep accounted for 48.20% during the post‐epidemic era. The work engagement of frontline nurses was at the medium level. Factors affecting nurses' sleep quality were the number of nurse night shifts, family support and nurse health. The factors affecting the nurse work engagement were monthly income, profession title, family support and self‐health status. There was a positive correlation between nurses' sleep quality and work engagement.

## 1 INTRODUCTION

Sleep is a physiological phenomenon in which organisms rest regularly. One‐third of human time is spent in sleep, which is one of human basic physiological needs (Dijk et al., 2010). Good sleep can restore individual energy, increase immunity, maintain brain metabolic balance, generate nerve impulses, improve cognition and emotion (Sandsmark et al., 2017), and promote mental health, life and work conditions (Li et al., 2018).

As a group with special professional characteristics, the profession of nursing have the characteristics of high work intensity, shift system, complex work content and need to be rigorous and careful and high work intensity. In recent 5 years, the literature reported that nurses generally had poor sleeping quality, with the incidence of sleeping problems of 46.32%–79.25%, and the total score of Pittsburgh sleep quality index (PQSI) was 7.13 ± 0.18 (95% CI: 6.78–7.50; Heath et al., 2019; Zhang et al., 2019; Zhao et al., 2020).

COVID‐19 is a disease important in public health globally which the World Health Organization later called coronavirus disease 2019 or COVID‐19 (Kim & Su, 2020; World Health Organization, 2020). Nurses have become a highly susceptible group due to direct contact with patients. According to the ICN, about 90,000 or 6% of all confirmed cases of COVID‐19 worldwide were health care workers (Labrague & de Los Santos, 2021). During the outbreak of the epidemic, the frontline clinical nurses, as the main forces in the fight against the epidemic, have psychological emergency reactions such as anxiety, depression and fear, which seriously affect the quality of sleep. The reasons for this phenomenon were due to the unknown development of the epidemic, strong infectivity, shortage of protective materials, infection of clinical medical staff, lack of experience in sudden infectious diseases, etc (Ayanian, 2020). The prevalence of sleep disorders among nurses has increased since the COVID‐19 outbreak (Salari et al., 2020). Meta‐analysis of 18 articles found that the prevalence of sleep disorders among nurses during the epidemic ranged from 12% to 87%, and the combined prevalence was estimated to be 43% (Al Maqbali et al., 2021).

Poor sleep quality affects the health of nurses, leads to obesity (Zhang, Chair, et al., 2020), causes varying degrees of damage to patients, leads to the loss of nurses' attention, alertness, concentration and judgement, leads to adverse events (Riemann et al., 2017)and threaten patients' safety (Park et al., 2018). At the same time, poor sleep quality is easy to reduce nurses' daytime work activity level and work efficiency (Chronister et al., 2015).

Work engagement is a kind of work‐related, persistent, diffuse, positive and fulfilling mental status, which is manifested in vigour, dedication and absorption (Schaufeli et al., 2002). It takes a.

central role in the JD‐R (Bakker & Demerouti, 2014). The status of work engagement is conducive to employees' efficient, happy and healthy work and has a positive impact on individual and organizational performance; Nurses' job involvement can affect nurses' job performance, and affecting nursing quality and patient safety. Before the COVID‐19, the researches showed that the state of nurses work engagement were at a medium level, which were reported in China (Wang et al., 2015), Spain (Orgambídez et al., 2019), the United States (Sullivan Havens et al., 2013) and Canada (Bamford et al., 2013). During the outbreak of COVID‐19, studies in China (Wang et al., 2020), Spain (Allande‐Cussó et al., 2021), Germany (Bernburg et al., 2021) and other countries found that nurses' work engagement showed a medium to high level, which might be due to the nature of job and awareness of the enormous importance of their work (Giménez‐Espert et al., 2020).

As to the variation of COVID‐19, although the transmission speed of virus is faster and faster, its pathogenicity has not increased, and the pressure on nurses is gradually reduced. At present, the epidemic situation in China is well controlled, and the anti‐epidemic has become a normalized management. In addition to undertaking daily nursing work, nurses also undertake the tasks of prevention of epidemic rebound, such as working in quarantine health observation, nucleic acid test, vaccination for COVID‐19, etc. Therefore, at a time when protective materials are relatively sufficient, the epidemic is better controlled, and epidemic prevention and control training is relatively in place, does nurses' sleep quality of improve, and what are the major influencing factors? Since the outbreak of COVID‐19, the nurses have been engaged in anti‐epidemic work, have their work engagement decreased, and what are the influencing factors? There are few articles on sleep quality and work engagement (Chu et al., 2021). Energetic and self‐regulatory resources have been proposed as mechanisms for the relationship between sleep and work engagement (Kühnel et al., 2016). Schleupner and Kühnel (2021) gathered data from 152 employees from diverse industries through an online survey. The results showed that sleep quality was positively related to work engagement. During the special period of the post‐epidemic era, is there any correlation between nurses' sleep quality and work engagement? These are the major concerns of this paper.

### 1.1 Aims

This study examined the status quo and influencing factors of sleep quality and work engagement of nurses participating in COVID‐19 during the post‐epidemic era and studied the relationship between them

## 2 METHODS

### 2.1 Study design

We conducted a cross‐sectional survey and applied correlational and predictive logic to determine the association between sleep quality and work engagement among nurses in Shanghai during the post‐epidemic era

### 2.2 Participants

A total of 1071 nurses who engaged in nursing tasks related to COVID‐19 in Shanghai, China, participated in the survey. The survey was conducted between June and November 2021. The participants were nurses from 11 medical institutions in Shanghai, including 2 tertiary hospitals, 1 secondary hospital, 7 community hospitals and 1 proprietary hospital. The inclusion criteria were as follows: ① had registered nurse's licence; ② participate in epidemic prevention and control related work within 1 month (such as working in quarantine health observation, nucleic acid test, vaccination for COVID‐19, nursing patients infected or suspected infected with COVID‐19, epidemiological investigation and health code verification, publicity for epidemic prevention and control, etc.) ③ informed consent and voluntarily participate in this study. The exclusion criteria were as follows: ① nurses unable to participate in the survey due to personal leave, sick leave, maternity leave and going out for further study or study; ② family members had been sick or dead in the past month; ③ suffering from diseases of central nervous system, urinary system, respiratory system and digestive system that affect sleep quality; ④ suffering from depression or other mental diseases

### 2.3 Ethical considerations

This survey was approved by the ethics committee of ‘REDACTED’ (ethics code: [2022] Review No. 193). The participants can fill in the questionnaire only after selecting the ‘agree’ button on the page before filling in the electronic questionnaire. During the filling process, they can choose to exit at any time without any information. The survey was conducted anonymously, and the personal confidentiality system was strictly implemented. After the questionnaire was collected, it was kept by the investigators in a unified manner. The privacy and personal safety information of the research subjects were strictly confidential.

### 2.4 Measures

For data collection, three questionnaires were given to each participant: a questionnaire collecting demographic data, the Pittsburgh Sleep Quality Index (PSQI) questionnaire and the Utrecht Work Engagement Scale‐9 (UWES‐9). The questionnaires were provided to the participants electronically through links. the home page uses unified guidelines to inform the purpose and significance of this study; the filling content of the questionnaire is set as required to avoid missing; select the drop‐down selection method for the object to fill in；to use the language familiar to the object to compile the questionnaire, so as to avoid the object having questions that cannot be answered; every IP can only fill the electronic questionnaire once to avoid repeated filling.

#### 2.4.1 Demographic data

This questionnaire was self‐designed after a literature review (Marvaldi et al., 2021; Qiu et al., 2020). The participants provided information about their sex, age, race, education background, BMI, hospital grade, positional title, years of work, personal monthly income, marital status, child status, type of work involved in the outbreak.

#### 2.4.2 Sleep quality

The Pittsburgh Sleep Quality Index (PSQI) was used, which was compiled by Buysse et al. (1989) and was used to evaluate the sleep status in the past 1 month. Liu et al. (1996) introduced it into China. It proves that there is good internal consistency, test–retest reliability, conception validity and empirical validity. The Cronbach's *α* was 0.845 (Li et al., 2005). The PSQI is a scored 18‐item self‐reported questionnaire, including 7 dimensions: subjective sleep quality, sleep time, sleep efficiency, sleep time, sleep medication, daytime function and sleep disorder. Each dimension is scored from 0 to 3 points, with a total score of 0 to 21 points. The PSQI total score of >7 points was used as the standard for sleep disorders in China (Liu et al., 1996). The higher the score, the worse the sleep quality. It was verified to be a standardized instrument with satisfactory reliability and validity. In this study, the Cronbach's alpha coefficient of the scale was 0.85.

#### 2.4.3 Work engagement

Work engagement was measured with Utrecht Work Engagement Scale‐9 (UWES‐9) developed.

By Schaufeli originally (Schaufeli et al., 2006) and contains three dimensions: vigour, dedication and absorption. There are 9 items in total. It was measured using g a seven‐point Likert scale ranging from 0 (never) to 6 (always). The higher the score, the better the work engagement. The Chinese version of the UWES‐9 was translated by Zhang and Gan (2005). The full score is 63 points, 9–27 points are low level work engagement, 28–45 points are medium level work engagement and 46–63 points are high level work engagement. The Chinese version of the UWES‐9 has good reliability and validity, which can be used to measure work engagement of employees in the Chinese context (Fong & Ng, 2012). In this study, the Cronbach's alpha coefficient of the scale was 0.932.

### 2.5 Data analysis

Univariate and bivariate descriptive data analysis was performed using the IBM SPSS Software (IBM). The measurement data are described by means and standard deviation. The counting data are described by frequency and composition ratio. Independent sample *t*‐test and *F*‐test were used to compare the difference of PSQI and UWES‐9 scores of different data. Pearson correlation analysis was used to explore the correlation between PSQI and UWES‐9. The odds ratios and 95% confidence intervals (CIs) were obtained by logistic regression. A *p*‐value < 0.05 was considered statistically significant.

## 3 RESULTS

### 3.1 Participants' characteristics

A total of 1071 questionnaires were issued which 1060 questionnaires were valid. The rate of validity of the questionnaires for this study was 98.97%. 11 participants had not answered the questions on scale and were, therefore, excluded from further analysis for better comparability. The ages of the participated nurses ranged from 20 to 54 years, with an average age of 31.85 ± 7.15 years, most of the participants were women (92.08%), the majority were Ethnic Han (97.45%), and 54.62% participants were married and had children. 74.15% of the participants had a bachelor's degree. BMI from 15.06 to 39.79, average 21.91 ± 3.11 kg/cm^2^. Most of participants (55.28%) worked in Tertiary hospitals and 51.04% of the participants' personal monthly income were >7000 CNY. Most of the participants were Junior nurse (66.89%). Engaged in Nursing time ranged from 1 to 36 years, with an average of 10.57 ± 7.77 years. A total of 47.17% nurses involved in epidemic prevention were temporarily secondment from other departments. The number of night shifts in the past month ranged from 0 to 24, with an average of 4.30 ± 4.57. About 85.38% family members supported participants in their outbreak‐related work. In addition, 50.19% participants reported their health as ‘GOOD’. The complete details of nurse characteristics are shown in Table 1.

**TABLE 1:** Participants' characteristics and comparison of sleep quality and work engagement (*n* = 1060).

Variabless	*N* (%)	PSQI score ($\bar{x}$ ± s)	*t/F*	*p*	UWES‐9 score ($\bar{x}$ ± s)	*t/F*	*p*
Sex			0.15	0.69			
Male	84 (7.92%)	7.24 ± 3.89			31.48 ± 13.08	0.29	0.59
Female	976 (92.08%)	7.39 ± 3.31			30.79 ± 10.95		
Age range, y			1.61	0.19		1.57	0.20
18–24	165 (15.57%)	7.21 ± 3.32			29.84 ± 11.31		
25–34	566 (53.40%)	7.48 ± 3.38			30.53 ± 11.11		
35–44	258 (24.34%)	7.09 ± 3.23			31.85 ± 10.98		
45–54	71 (6.69%)	7.38 ± 3.35			32.00 ± 11.29		
Race			0.65	0.42		0.99	0.32
Ethnic Han	1033 (97.45%)	7.36 ± 3.36			30.90 ± 11.17		
Others	27 (2.55%)	7.89 ± 3.14			28.74 ± 9.43		
Marital status			1.30	0.27		1.64	0.18
Unmarried	349 (32.92%)	7.58 ± 3.45			29.86 ± 10.94		
Married and unbred	117 (11.04%)	7.62 ± 3.40			31.64 ± 11.21		
Married and bred	579 (54.62%)	7.19 ± 3.31			31.19 ± 11.17		
Divorce or widowed	15 (1.42%)	7.73 ± 2.34			33.73 ± 12.68		
Education			0.16	0.92		1.36	0.25
Polytechnic school	10 (0.94%)	6.80 ± 4.85			29.90 ± 12.44		
Junior college	253 (23.87%)	7.46 ± 3.32			29.71 ± 10.45		
Bachelor	786 (74.15%)	7.35 ± 3.35			31.18 ± 11.31		
Master	11 (1.04%)	7.45 ± 3.70			33.64 ± 11.45		
BMI, kg/cm^2^			1.61	0.20		2.98	0.05
≤18.4	95 (8.96%)	7.88 ± 3.28			28.22 ± 9.98		
18.5–23.9	723 (68.21%)	7.38 ± 3.32			31.02 ± 10.86		
≥24.0	242 (22.83%)	7.16 ± 3.47			31.34 ± 12.20		
Hospital level			1.19	0.31		2.14	0.09
Proprietary hospital	10 (0.95%)	7.40 ± 3.02			33.20 ± 9.98		
Community hospital	237 (22.36%)	7.39 ± 3.26			29.53 ± 11.06		
Secondary hospital	227 (21.42%)	7.73 ± 3.41			30.34 ± 10.96		
Tertiary hospital	586 (55.28%)	7.23 ± 3.37			31.52 ± 11.20		
Personal monthly income, CNY			1.59	0.19		11.03	0.00
<3000	16 (1.51%)	7.37 ± 3.24			24.87 ± 10.58		
3000–5000	150 (14.15%)	7.58 ± 3.51			28.65 ± 11.78		
5001–7000	353 (33.30%)	7.62 ± 3.26			29.25 ± 11.10		
>7000	541 (51.04%)	7.16 ± 3.37			32.66 ± 10.67		
Profession title			0.93	0.39		8.52	0.00
Junior nurse	709 (66.89%)	7.47 ± 3.34			29.91 ± 11.20		
Intermediate nurse	313 (29.53%)	7.20 ± 3.43			32.46 ± 10.82		
Senior nurse	38 (3.58%)	7.03 ± 3.07			34.95 ± 9.97		
Years of work experience			0.72	0.58		1.43	0.22
<5	251 (23.68%)	7.26 ± 3.50			30.26 ± 11.52		
5–9	291 (27.45%)	7.47 ± 3.14			30.00 ± 11.46		
10–19	345 (32.55%)	7.27 ± 3.45			31.54 ± 10.83		
20–29	141 (13.30%)	7.73 ± 3.18			32.09 ± 9.69		
≥30	32 (3.02%)	6.97 ± 3.82			29.94 ± 13.60		
Participation in work related to epidemic situation in the last month			1.16	0.33		2.08	0.07
Nucleic acid test	37 (3.49%)	7.35 ± 3.10			31.51 ± 12.26		
Vaccination	292 (27.54%)	7.27 ± 3.36			31.45 ± 10.84		
Working in quarantine health observation	11 (1.04%)	5.73 ± 2.24			33.09 ± 14.51		
Hospital fever clinic	17 (1.60%)	6.82 ± 3.17			37.12 ± 9.48		
Epidemiological inspection at hospital entrance and wards	499 (47.08%)	7.35 ± 3.44			30.73 ± 11.18		
Multiple positions	203 (19.15%)	7.74 ± 3.25			29.40 ± 10.95		
Job nature			2.72	0.07		5.45	0.00
Temporary secondment (less than 1 month)	500 (47.17%)	7.62 ± 3.40			31.58 ± 10.73		
Fixed position	432 (40.75%)	7.11 ± 3.33			29.52 ± 11.34		
Long‐term secondment (more than 1 month)	128 (12.08%)	7.33 ± 3.20			32.40 ± 11.56		
Number of night shifts			15.26	0.00		0.53	0.59
0	378 (35.66%)	6.72 ± 3.33			30.87 ± 11.96		
1–5	302 (28.49%)	7.35 ± 3.17			31.32 ± 11.05		
≥6	380 (35.85%)	8.05 ± 3.40			30.43 ± 10.32		
Family member support			24.12	0.00		14.14	0.00
Support	905 (85.38%)	7.09 ± 3.28			31.58 ± 11.13		
Part of the support	147 (13.87%)	9.09 ± 3.24			26.44 ± 10.02		
Non‐support	8 (0.75%)	8.63 ± 4.72			27.75 ± 12.53		
Self‐health status			92.11	0.00		48.36	0.00
Poor	38 (3.58%)	10.84 ± 3.41			21.47 ± 10.61		
Fair	490 (46.23%)	8.42 ± 2.98			28.36 ± 9.47		
Good	532 (50.19%)	6.17 ± 3.18			33.80 ± 11.66		

### 3.2 Sleep quality

It was found that the average PSQI score of the nurses was 7.37 ± 3.54 points during the post‐epidemic era. Many nurses (*n* = 511, 48.20%) reported poor sleep quality (PSQI > 7). Daytime dysfunction was the highest (M = 1.82, SD = 0.98), followed by Sleep duration (M = 1.37, SD = 0.77; Table 2).

**TABLE 2:** Descriptive statistics of sleep quality index scores (*n* = 1060).

Variables	Score (mean ± SD)
Subjective sleep quality	1.25 ± 0.75
Sleep latency	1.26 ± 0.85
Sleep duration	1.37 ± 0.77
Habitual sleep efficiency	0.43 ± 0.77
Sleep disturbances	1.06 ± 0.61
Use of sleeping medications	0.19 ± 0.55
Daytime dysfunction	1.82 ± 0.98
PSQI score	7.37 ± 3.54

### 3.3 Work engagement

The study found that the average UWES‐9 score of the nurses was 30.84 ± 11.13 points, which was a medium degree of work engagement. Absorption was the highest (M = 10.44, SD = 3.89), followed by Vigour (M = 10.23, SD = 3.78; Table 3).

**TABLE 3:** Descriptive statistics of work engagement index scores (*n* = 1060).

Variables	Score (mean ± SD)
Vigour	10.23 ± 3.78
Dedication	10.17 ± 4.06
Absorption	10.44 ± 3.89
UWES‐9 score	30.84 ± 11.13

### 3.4 Factors predicting sleep quality

Univariate outcome analysis found that the number of night shifts, family support and health status of nurses affected their sleep quality with statistical difference (*p* < 0.05). Among them, the sleep quality of non‐shift nurses was higher than that of shift nurses, and the sleep quality of nurses with family support was higher than that of nurses with partial family support and non‐support. The sleep quality of healthy nurses was higher than that of fair or poor health nurses (Table 1).

Results of multivariate regression using total score of PSQI as the dependent variables and items meaningful in the univariate analysis were included as independent variables. Those showing statistically significant association were number of night shifts (*β* = 0.167, *p* < 0.001), family member support (*β* = 0.106, *p* < 0.001), self‐rated health (*β* = 0.360, *p* < 0.001; Table 4).

**TABLE 4:** Multivariate regression for variables associated with PSQI (*n* = 1060).

Variable	*B*	SE	*β*	*t*	*p*
Number of night shifts	0.661	0.110	0.167	6.004	<0.001
Family member support	0.937	0.252	0.106	3.717	<0.001
Self‐health status	−2.130	−0.360	0.360	−12.558	<0.001

*Note*: *R* ^2^ = 0.188, Adj *R* ^2^ = 0.185, *F* = 81.391, *p* < 0.001.

### 3.5 Factors predicting work engagement

Univariate outcome analysis found that the average monthly income, profession title, job nature, family support and health status of nurses who participated in the work related to the epidemic situation affected their work engagement, with statistical difference (*p* < 0.05). Among them, the work engagement of nurses with income >7000 CNY was higher than that of other income groups. The work engagement of nurses with senior titles was higher than that of nurses with intermediate titles and junior titles. The work engagement of nurses with long‐term secondment was higher than that of temporary secondment and fixed position. The work engagement of nurses who received family support was higher than that of nurses who received partial family support and no family support. The work engagement of nurses with good health was higher than that of nurses with fair or poor health (Table 1).

Results of multivariate regression using total score of UWES‐9 as the dependent variables and items meaningful in the univariate analysis were included as independent variables. Those showing statistically significant association were personal monthly income (*β* = −0.115, *p* < 0.001), profession title (*β* = 0.127, *p* < 0.001), family member support (*β* = −0.084, *p* < 0.001), self‐rated health (*β* = 0.261, *p* < 0.001; Table 5).

**TABLE 5:** Multivariate regression for variables associated with UWES‐9 (*n* = 1060).

Variable	*B*	SE	*β*	*t*	*p*
Personal monthly income	−1.654	0.422	−0.115	−3.921	<0.001
Profession title	2.556	0.586	0.127	4.360	<0.001
Post nature	−0.401	0.470	−0.025	−0.854	0.393
Family member support	−2.451	0.869	−0.084	−2.819	0.005
Self‐rated health	5.125	0.591	0.261	8.667	<0.001

*Note*: *R* ^2^ = 0.124, Adj *R* ^2^ = 0.120, *F* = 29.788, *p* < 0.001.

### 3.6 Correlation analysis

Pearson rank correlations were calculated for Sleep quality and Work engagement. The results showed that a negative correlation was found between PSQI and UWES‐9 the total score and their dimensions during the post‐epidemic era (*p* < 0.05; Table 6).

**TABLE 6:** Pearson correlations between sleep quality and work engagement.

	SQ1	SQ2	SQ3	SQ4	SQ5	SQ6	SQ7	GSQ	Vigour	Dedication	Absorption
SQ1											
SQ2	0.526**										
SQ3	0.3618**	0.283**									
SQ4	0.232**	0.206**	0.404**								
SQ5	0.499**	0.399**	0.205**	0.123**							
SQ6	0.270**	0.220**	0.118**	0.074**	0.182**						
SQ7	0.528**	0.303**	0.319**	0.152**	0.484**	0.173**					
GSQ	0.782**	0.683**	0.624**	0.506**	0.641**	0.407**	0.714**				
Vigour	−0.228**	−0.165**	−0.091**	−0.078*	−0.191**	−0.073*	−0.271**	−0.258**			
Dedication	−0.168**	−0.160**	−0.038	−0.039	−0.131**	−0.063*	−0.169**	−0.174**	0.864**		
Absorption	−0.225**	−0.160**	−0.086**	−0.070*	−0.181**	−0.071*	−0.252**	−0.245**	0.863**	0.821**	
UWES‐9	−0.218**	−0.164**	−0.076**	−0.066*	−0.177**	−0.073*	−0.243**	−0.238**	0.957**	0.943**	0.945**

Abbreviations: GSQ, global sleep quality; SQ1, Subjective sleep quality; SQ2, sleep latency; SQ3, sleep duration; SQ4, habitual sleep efficiency; SQ5, sleep disturbances; SQ6, use of sleeping medication; SQ7, daytime dysfunction.

* *p* < 0.05.

** *p* < 0.01.

## 4 DISCUSSION

The present cross‐sectional study provided descriptive information about sleep quality and work engagement, and statistically significant factors that contributed to poor sleep quality and work engagement among nurses during the post‐epidemic era.

The total PSQI score of frontline nurses was (7.37 ± 3.54). The sleep quality of nurses was not optimistic in China during the post‐epidemic era, but it was better than that of frontline nurses (7.96 ± 2.12) during the outbreak of COVID‐19 (*t* = − 5.68, *p* < 0.01; Wu et al., 2020). It still was worse than that of Chinese nurses' sleep quality (6.72 ± 3.27) during the non‐epidemic period (*t* = 6.35, *p* < 0.01; Liu & Chen, 2015). This was due to the gradual understanding of COVID‐19 by medical personnel, the reduction of fear of COVID‐19 and the sufficient protective materials, which lead to the improvement of nurses' sleep quality compared with the outbreak of the epidemic. However, at this stage, nurses are facing the pressure of the outbreak of the epidemic again. The continuous high‐pressure state will cause changes in the cortisol level of nurses day and night, cause sleep disorders (Vargas et al., 2018) and lead to poor sleep quality compared with the normal group. It is recommended that managers should pay attention to assessing the sleep quality of nurses and take measures to improve it. The two dimensions of daytime dysfunction and sleep duration were higher. Nurses' shift work is necessary to achieve timely, effective and safe patient care without interruption (Park et al., 2018). The circadian rhythm disorder caused by shift work (Chaiard et al., 2019), irregular work and rest, and overtime may be the reason for the high scores of sleep time and daytime dysfunction. The study found that the higher the level of mindfulness of nurses during the COVID‐19 epidemic, the better the quality of sleep (Yin et al., 2021). It was proposed that mindfulness therapy could regulate the individual's cognition of the environment and state, and consciously identify self‐thoughts, thoughts and other aspects, so as to promote the improvement of sleep quality (Kemper et al., 2015). Therefore, it is suggested that through mindfulness based training, nurses should pay more attention to the work of epidemic prevention, reduce the negative emotions and work pressure and take active response measures to effectively improve the sleep quality of nurses.

The number of night shift affected nurses' quality of sleep. This study investigated the worse the nurse sleep quality when they worked more than five night shifts per month. The more frequent the night shift, the worse the sleep quality (Dong et al., 2017). The prevalence of sleep disturbance among shift nurses is three‐to‐eight times higher than that in the general population (Ohayon, 2002). Because the night shift workers sleep in the daytime, the light and the quiet degree of the environment will interfere with sleep during the daytime, resulting in poor sleep quality. Most nurses are unable to adjust their circadian rhythm according to their atypical sleep and wakefulness duration, and they seem to be prone to excessive sleepiness and/or insomnia (d'Ettorre et al., 2020). Shift work is inevitable for nurses, but it is necessary to find better shift‐work pattern to improve sleep quality (Park et al., 2018). Managers can optimize the allocation of nurses' human resources, reduce the frequency of night shift, allow nurses to ‘work less and no overtime’ and have reasonable rest time, so as to improve the quality of sleep and promote the physical and mental health of clinical nurses.

This finding also found that the nurses who received family support for anti‐epidemic work had better sleep quality. The improvement of family support can reduce nurses' psychological problems such as depression and anxiety. Therefore, the hospital administrator should guide the nurses to balance the relationship between work, family and life and take corresponding measures to obtain their family's understanding and support for the anti‐epidemic work when necessary.

The results also showed that the health status affects the sleep quality of nurses. The worse the health status, the worse the sleep quality. The physical condition of medical personnel is negatively correlated with fatigue (Lin et al., 2017). As the epidemic has increased the nursing workload, the poor physical condition will reduce the work efficiency, lead to the extension of working hours and also increase the work pressure, and eventually the sleep quality becomes poor. It is suggested that hospital managers should consider nurses' physical condition when selecting nurses to take charge of the epidemic work, so as to avoid affecting the quality of the epidemic work due to poor sleep.

The average score of UWES‐9 of nurses during the post‐epidemic era was 3.43 (SD = 1.23), which was at the middle level, similar to the results of Zhang and Zheng (2016). In the epidemic outbreak period, Zhang, Zhao, et al. (2020) surveyed 242 nurses in Wuhan and found that their work engagement score was 4.83 (SD = 1.01). People do not feel tired in the early stage of work and have a high enthusiasm for work participation (Spinetta et al., 2000). However, nurses are prone to burnout in the work process, leading to the gradual shift of energy to exhaustion, Active participation turns to estrangement and effectiveness turns to ineffectiveness (Maslach et al., 2001). With the continuous progress of epidemic prevention, it is suggested to increase nurses' work engagement to prevent burnout. In the post‐epidemic era, the scores of each dimension of work engagement from high to low are absorption, vigour and dedication. Absorption refers to the state in which nurses concentrate on their work, immerse themselves in the achievements and happiness brought by work, feel that time flies and are unwilling to get away from work. The reason is that nurses are the main force of epidemic prevention and have a strong sense of mission and value. Vigour means that nurses are full of energy and good mental toughness in their work. They are willing to invest more time and energy in the anti‐epidemic work. They can face difficulties actively and persevere. This is due to the high recognition of nurses by the whole society during the epidemic period, which virtually increases nurses' sense of professional achievement and motivation to devote themselves to work. Dedication refers to that nurses devote themselves to the anti‐epidemic work, feel the significance of the work, are willing to accept challenging work and generate psychological satisfaction. The score of this dimension was relatively low, which might be related to the fact that the anti‐epidemic work has become a normal work after 2 years of the epidemic. The challenge gradually subsided in the long‐term work, and the nurses were relatively young in this study. Managers should pay attention to the current situation of insufficient work engagement of clinical nurses during the epidemic period, take scientific and effective measures to enhance the work engagement of nurses under public health emergencies, ensure the quality of epidemic prevention and promote the stable development of nursing teams.

In this study, we found that the professional title of nurses affects the work engagement of nurses, and the higher work engagement of nurses with senior titles. Nurses with higher professional titles can still maintain positive emotions in difficult working environment, their self‐realization and work expectation are higher than those with lower professional titles, and their social respect and recognition and social status identification needs are higher than those of nurses with lower professional titles. They can fully work and live according to their own values, constantly reflect on themselves, and make positive adaptive adjustments to nursing work, Correctly understand the significance of nursing work, so as to truly experience and maintain long‐term work engagement (Han & Wang, 2017).

Family support, average monthly income and health status of nurses affected nurses' work engagement. In the collectivism and family oriented Asian culture, the family is the main source of support (Matsumoto et al., 2017). Lu et al. (2011) and other researchers found that nurses can get more family support, and nurses' work engagement will be higher, especially in the high‐intensity work state, supporting the theoretical model of work‐family enrichment (Demerouti et al., 2010). Performance pay refers to the reward given to employees for their work performance. As an important factor affecting nurses' job satisfaction, salary satisfaction can stimulate their work enthusiasm and thus affect their work engagement level (McHugh & Ma, 2014). It is suggested that managers should pay attention to the important role of salary satisfaction in improving nurses' work engagement, and formulate individualized salary levels and increase other forms of non‐wage benefits according to the nature of the post, work intensity and risk of the epidemic. Health is divided into physical health and mental health. Poor health may lead to decreased productivity, poor work performance and increased absenteeism (Alavinia et al., 2009). Nursing managers should take effective measures to improve the mental health of nurses and improve the level of work engagement.

The results of this survey showed that the sleep quality of nurses was related to their work engagement during the post‐ epidemic era. The results showed that a negative correlation was found between PSQI and UWES‐9 the total score and their dimensions (*p* < 0.05), that is, the worse the sleep quality, the worse the work commitment, which was the same as the research results of Chu et al. (2021) and Ricarda Schleupner and Kühnel (2021). The sleep quality of employees can directly affect work engagement and can also indirectly affect work engagement through mental health as an intermediary (Schleupner & Kühnel, 2021). This view is also consistent with JD‐R framework and the effort recovery model. Sleep problems will affect the recovery of energy and physical strength of personnel and affect their work engagement. Meanwhile, people with poor sleep quality will affect their mood, cause anxiety and worry, and lead to physical and mental fatigue, thus affecting their work engagement.

## 5 LIMITATIONS AND RECOMMENDATIONS FOR FUTURE RESEARCH

This study has some limitations. First of all, the participants included in the study were all from Shanghai. As China's international economy, finance, trade, shipping and national centre, Shanghai has a large flow of people, and there are certain difficulties and pressures in epidemic prevention and control. The participants of the study are representative to certain extents. Therefore, future studies may provide more general results, if their studies can involve nurses from other regions of the country. Secondly, this study is a cross‐sectional study. Future studies should explore the Organizational factors that may affect the sleep quality and work engagement of nurses in the post‐epidemic era (such as the adequacy of hospital resources, staffing level, etc.).

## 6 IMPLICATIONS FOR NURSING MANAGEMENT

Compared with the outbreak period, the sleep quality of nurses has improved, but their work engagement has decreased during the post‐epidemic era. Hospital managers should pay great attention to the sleep problems and work engagement of current anti‐epidemic nurses, realize the mutual influence of sleep quality and work engagement, and do a good job of adjusting the sleep problems and work engagement of nurses from multiple ways and perspectives. The measures include (1) timely carry out the assessment of the sleep quality of frontline nurses, strengthen psychological assistance measures and reduce stress; (2) optimize the allocation of nurses' human resources and reduce the frequency of night shift; (3) the frontline nurses in the fight against epidemic disease regularly carry out the learning of recovery experience and lead the managers to improve the ability of recovery experience of nurses from four aspects of psychological detachment, relaxation experience, mastery experience and control experience, so as to alleviate the sleep problems, enhance work engagement (Badu et al., 2020); (4) fixed nursing staff for epidemic prevention work posts; (5) select physical health nurses to participate in the epidemic work; (6) improving family and social care and support, and good social and family support can help to improve sleep quality and work engagement.

## 7 CONCLUSION

This study found that the sleep quality of frontline nurses was generally hindered, and their work engagement was at a medium level during the post‐epidemic era. The sleep quality of nurses was correlated with their work engagement. Nursing managers need to take corresponding measures to improve the nurses' sleep quality and work engagement. At the same time, it also provides reference for nursing managers to maintain the physical and mental health of nursing staff and improve the epidemic protection and nursing work quality during the post‐epidemic era.

## FUNDING INFORMATION

This was funded by Shanghai Pudong New Area Caring for Nurses Project (grant no. PW2021‐HZ03), Academic Leaders Training Program of Pudong Health Bureau of Shanghai (grant no. PWRd2022‐16), Important Weak Subject Construction Project of Shanghai Pudong New Area Health Commission (grant no. PWZbr2022‐04).

## CONFLICT OF INTEREST STATEMENT

The authors declare no conflicts of interest.

## ETHICS STATEMENT

This study received Research Ethics Committee approval from accreditation of the Ethics Committee of the ‘REDACTED’.

## Data Availability

The data that support the findings of this study are available from the first author, [author Zhuang Huiren], upon reasonable request.
